# Radical Resection and Reconstruction of Recurrent Periocular Basal Cell Carcinoma Involving Orbit and Midface: A Multidisciplinary Case Report

**DOI:** 10.7759/cureus.90373

**Published:** 2025-08-18

**Authors:** Amir Kaboodrangi, Andy Aleman Espino, Nicholas K Lago, Michael Jonczyk, George Dreszer

**Affiliations:** 1 Graduate Medical Education, Broward Health Medical Center, Fort Lauderdale, USA; 2 College of Osteopathic Medicine, Nova Southeastern University Dr. Kiran C. Patel College of Osteopathic Medicine, Fort Lauderdale, USA; 3 General Surgery Residency, Broward Health Medical Center, Fort Lauderdale, USA; 4 Plastic and Reconstructive Surgery, Broward Health Medical Center, Fort Lauderdale, USA; 5 Plastic Surgery, Broward Health Medical Center, Fort Lauderdale, USA

**Keywords:** basal cell carcinoma, calvarial bone graft, facial reconstruction, head and neck surgery, maxillectomy, microvascular surgery, orbital exenteration, periocular malignancy, radial forearm free flap, skin cancer recurrence

## Abstract

Basal cell carcinoma (BCC) is a locally invasive skin malignancy that can affect the periocular region, often posing significant reconstructive challenges when recurrent or inadequately treated. We report the case of an 80-year-old male with a history of multiple facial BCC excisions who presented with progressive left facial tissue loss and vision loss. Biopsy confirmed recurrent BCC, and positron emission tomography computed tomography (PET-CT) revealed a hypermetabolic mass involving the left orbit, zygoma, and maxillary sinus without distant metastasis. The patient underwent radical oncologic resection, including orbital exenteration, maxillectomy, lateral rhinotomy, ethmoidectomy, and resection of the zygoma and pterygopalatine fossa. Reconstruction involved a split calvarial bone graft for skeletal support and a radial forearm free flap for soft tissue coverage. Pathology showed a 4.7 cm tumor with invasion into conjunctiva, muscle, and bone, and involvement of the deep margin. He recovered well, received adjuvant radiation therapy, and later underwent revision surgery for hardware exposure, with no malignancy identified on pathology. This case illustrates the importance of a multidisciplinary approach and long-term follow-up in managing complex, recurrent periocular BCC to optimize oncologic control and reconstructive outcomes.

## Introduction

Basal cell carcinoma (BCC) is the most prevalent form of nonmelanoma skin cancer worldwide. Due to underreporting in cancer registries, the true incidence remains uncertain, but it is estimated that BCC affects approximately 2 million Americans annually, more than all other cancers combined [1]. The leading etiologic factor is ultraviolet light exposure, and lesions frequently appear on the head and neck, including the periocular region [1,2]. In Caucasians, BCCs account for approximately 90% of eyelid malignancies [3].

Although BCCs typically exhibit slow growth and rare metastatic potential (<1%), they can infiltrate local structures extensively if inadequately treated or recurrent [1]. Up to 20% of periocular BCCs recur after excision, and recurrences often carry a worse prognosis due to deeper tissue involvement and complex anatomy [3]. In aggressive cases involving perineural or orbital invasion, recurrence may necessitate radical surgical approaches to achieve disease control.

Current guidelines recommend histologic confirmation via skin biopsy, preferably including a portion of the dermis, to classify histologic subtypes and determine aggressiveness [4,5]. In cases of suspected deep invasion, cross-sectional imaging is critical to delineate tumor extent and inform surgical planning [1].

Mohs micrographic surgery (MMS) remains the gold standard for periocular BCCs due to tissue-sparing precision and a low recurrence rate [1]. However, wide local excision or en bloc resection followed by postoperative margin evaluation remains an acceptable approach, especially in cases where Mohs surgery is not readily available, and if chosen as the surgical treatment, margins of 8mm are recommended to achieve a complete resection of the tumor [6].

Though a wider resection is associated with higher morbidity and the need for advanced reconstructive techniques, in such scenarios, reconstructive strategies may involve free tissue transfer using vascularized bone and/or soft tissue flaps, tailored to the extent of resection and defect location [7,8].

In this paper, we present the uncommon case of a patient with recurrent BCC and extensive bony involvement of the orbit and midface that required the use of a multidisciplinary surgical strategy that combined split calvarial bone grafting and a radial forearm free flap for reconstruction. These techniques are not commonly reported in the literature for periocular BCC and underscore the surgical complexity involved in managing locally advanced facial tumors.

## Case presentation

An 80-year-old man with a known history of biopsy-proven BCC of the left face presented to our head and neck oncology clinic for evaluation of a progressively enlarging defect and worsening vision in his left eye. He had undergone multiple wide local excisions with local reconstructions in the past. On examination, there was a visibly expanding defect in the left midface with periorbital involvement. Visual acuity in the left eye was limited to light perception only. A recent biopsy performed by his otolaryngologist confirmed recurrent BCC.

**Figure 1:**
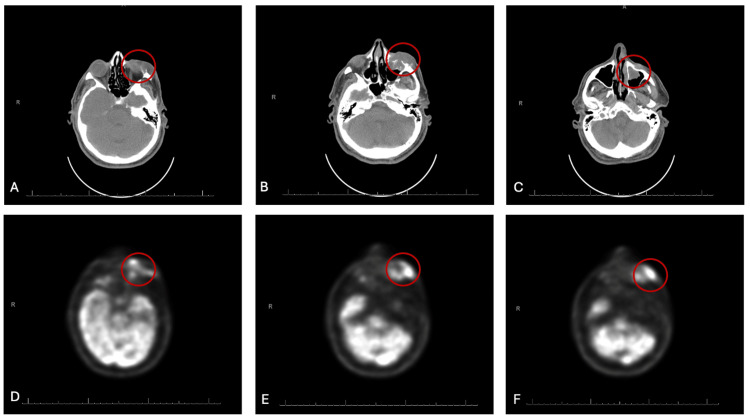
PET-CT of the Head. High-grade, hypermetabolic (D, E, F), infiltrative mass (A, B, C) centered on the left orbit, extending into the maxillary sinus, with destruction of mid and lower orbital bones and both superficial and deep soft tissue involvement.

A positron emission tomography computed tomography scan (PET-CT scan) of the head revealed a high-grade, hypermetabolic, infiltrative mass centered on the left orbit, extending into the maxillary sinus, with destruction of mid and lower orbital bones and both superficial and deep soft tissue involvement (Figure 1). There was no evidence of metastasis to the neck or chest.

After a comprehensive discussion of treatment options with the patient, a decision was made to proceed with radical oncologic resection. The patient was also evaluated by our reconstructive surgery team in anticipation of complex soft tissue and bony reconstruction. Radiation oncology was consulted for adjuvant therapy planning.

Oncologic resection

Once in the operating room, the left periorbital cheek, orbit, and nasal cavity were marked to ensure adequate margins. The incision was made and carried down to the bone circumferentially around the marked area. It was a difficult dissection secondary to significant inflammatory change and desmoplastic reaction from both the malignancy and previous operations.

The tumor appeared to extend posteriorly through the temporalis muscle, and it also appeared to be involving the zygoma; therefore, we performed a radical resection of this bone utilizing osteotomes and a sagittal saw. The infraorbital nerve was grossly involved with the tumor and followed posteriorly along its course to the infratemporal fossa, ligated, and excised. The intraorbital contents were then dissected in the subperiosteal plane and followed down circumferentially. The optic nerve was identified and ligated as proximally as possible at its entry into the orbital cavity. This completed the orbital exenteration portion of the procedure.

The tumor was grossly invading through the maxillary bone below the level of the lamina papyracea with involvement of the soft tissue of the ethmoid. Therefore, a total ethmoidectomy was performed. A lateral rhinotomy was then performed, providing access to the medial maxilla and nasal cavity. We then proceeded with the maxillectomy, ensuring inclusion of the maxillary contents and preserving the hard palate. The sharp bony edges were smoothed, and the wound bed was copiously irrigated. Final inspection of the surgical defect revealed gross removal of the tumor, which included the following: full-thickness periorbital soft tissue, zygoma bone, pterygopalatine fossa contents, lateral nasal cavity, ethmoid sinus, maxillary sinus, and orbit.

Reconstruction

**Figure 2:**
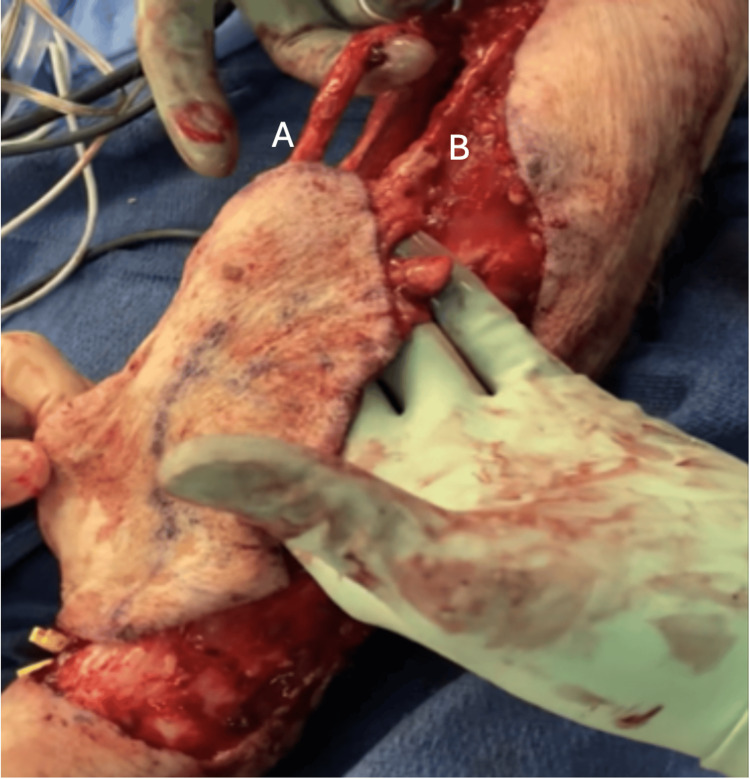
Left radial forearm free flap with two vessels, the radial artery and cephalic vein, entering the skin paddle. A) Radial artery; B) cephalic vein

We proceeded with a left radial forearm free flap to the left face. The flap was elevated under tourniquet control with dissection of a 12 × 10 cm skin paddle (Figure 2). Dissection of the radial vessels began proximally between the brachioradialis and the flexor carpi radialis. The bifurcation of the brachial artery was identified in the antecubital fossa. 

**Figure 3:**
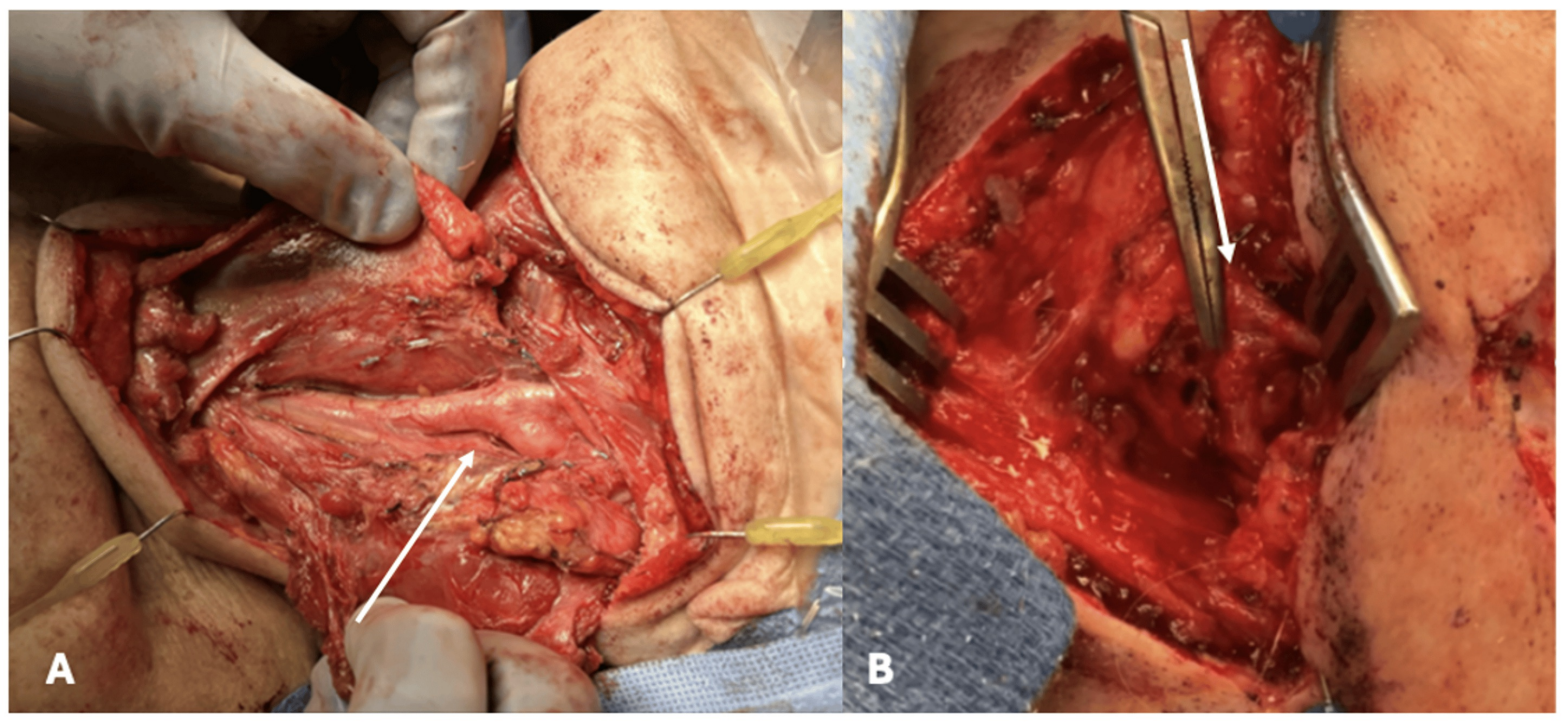
Exposure of the common carotid artery. The carotid artery at its bifurcation is indicated by the arrow in A, the facial artery, coming as a branch of the external carotid artery, is indicated by the arrow in B.

Synchronous dissection occurred in the patient’s neck to identify the facial vessels (Figure 3). An incision was made, and the subcutaneous tissues were divided until the facial artery was identified, coming from the external carotid artery. However, the flow was minimal. Therefore, we performed a Fogarty catheter embolectomy of the facial artery, which produced great pulsatile arterial flow. 

**Figure 4:**
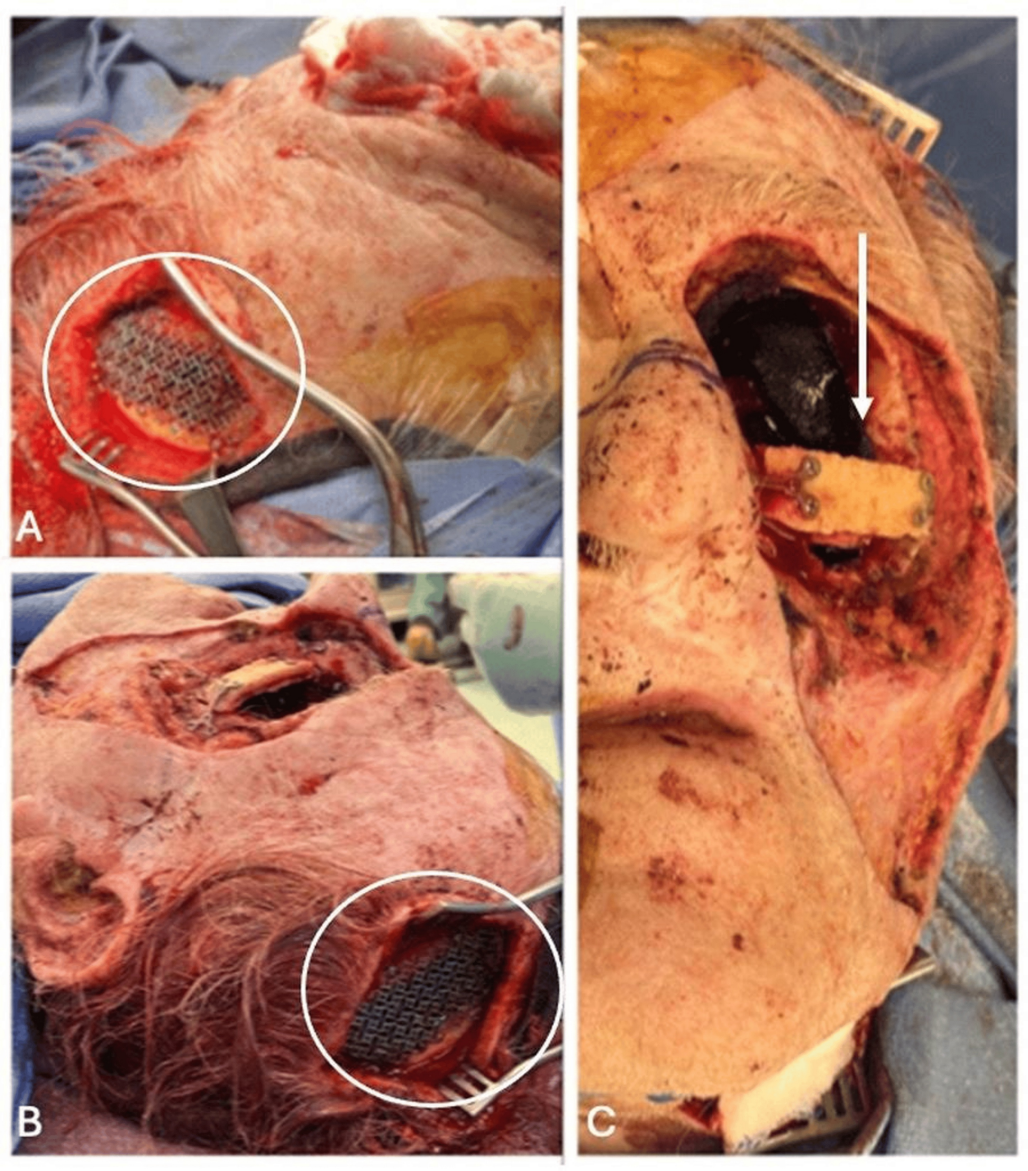
The two left images (A and B) depict the titanium mesh used to repair the calvarial defect from the split calvarial bone graft. The right-sided image (C) shows the extent of oncologic resection along with the reconstruction of the zygoma using the calvarial autograft. Titanium mesh in images A and B within the circle. Reconstruction of the zygoma using the calvarial autograft in image C is indicated by the arrow.

Next, we transitioned our focus to the maxillary bone reconstruction (Figure 4). An incision was made at the patient’s left occiput, and we dissected into the calvarium to harvest a bone graft without violation of the internal table. A titanium mesh was used to repair the calvarial defect with titanium screws. This bone graft was then inset, reconstructing the patient's infraorbital rim and the zygomatic arch. 

**Figure 5:**
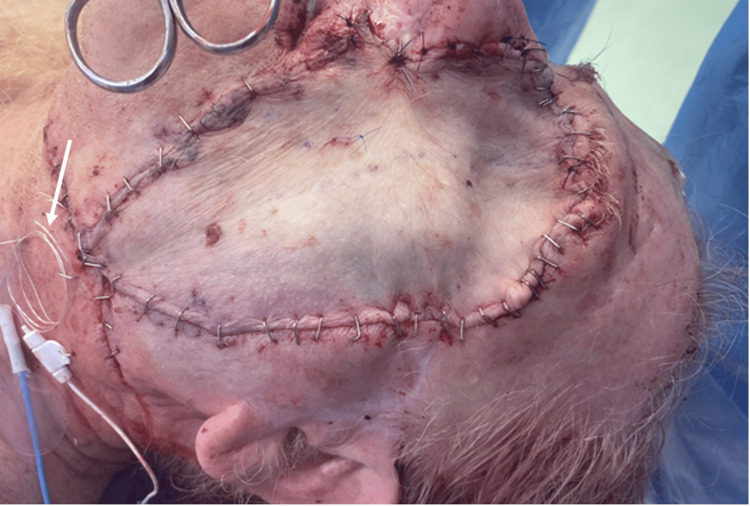
Depiction of the surgical site at the end of the operation with the radial forearm free flap sized to fit the defect and secured with surgical staples and Prolene sutures. The implanted microvascular Doppler is seen exiting the inferior surgical site, pointed by the arrow.

We then proceeded with the anastomosis and inset of our free flap. The radial artery and cephalic vein were ligated proximally near the bifurcation of the brachial artery. A microvascular anastomosis to the facial artery and vein was performed, and an implantable microvascular Doppler was inserted and confirmed to have adequate flow (Figure 5). A drain was placed, and the flap was secured with a combination of interrupted Prolene sutures and surgical staples. 

Pathology and outcome

Final pathology revealed a 4.7 cm BCC with invasion into conjunctiva, skeletal muscle, and bone. Depth of invasion was at least 1.7 cm with involvement of the deep surgical margin. There was extensive bone invasion into the maxilla and zygoma. The submandibular lymph node was negative for malignancy. Final staging was pT4bN0M0.

The patient tolerated surgery well and was discharged on postoperative day 8. He received adjuvant radiation therapy to the face following his initial surgery. Nine months later, he returned to our facility with complaints of hardware exposure from both the calvarial donor site and the left cheek reconstruction. Examination revealed erosion at the site of the split calvarial bone graft with exposed hardware, and early exposure of hardware in the left midface region. The previously placed flap was viable with appropriate blood flow. He was subsequently taken for operative debridement, re-advancement of the previous free flap, removal of hardware from the scalp and cheek, and reconstruction of both defect sites. Surgical pathology from intraoperative specimens showed no evidence of malignancy. He was seen in subsequent follow-up and was reported to be healing well without complications.

## Discussion

This case exemplifies the challenges and considerations involved in managing recurrent periocular BCC with deep orbital and midfacial invasion. After discussion with our surgical team and the patient, radical surgical excision was chosen as the surgical treatment. Although MMS is well established for primary and low-risk periocular BCCs, offering tissue preservation and low recurrence rates [2], wide local excision followed by postoperative margin evaluation remains an acceptable approach, especially in cases where Mohs surgery is not readily available [6].

Similarly, radiation monotherapy was deemed suboptimal in this scenario. While radiation may achieve disease control in selected patients or as adjuvant therapy, its efficacy as a primary modality is limited in extensive, deeply infiltrative tumors. Scar formation may also impede early recognition of recurrence [9,10].

Reconstruction of the resulting complex defect required multidisciplinary planning. We elected to use a split calvarial bone graft for osseous reconstruction of the infraorbital rim and zygomatic arch due to its reliable osteogenic properties, proximity to the surgical field, and lower donor site morbidity [11]. Alternatives such as fibula free flaps offer robust bone stock and can support prosthetic rehabilitation but are associated with longer operative time, increased morbidity, and functional limitations [12]. The radial forearm free flap was chosen for its thin, pliable skin, consistent vascular anatomy, and suitability for facial contouring. While anterolateral thigh (ALT) flaps provide more volume and have less donor site morbidity, they may be bulkier and more difficult to contour for delicate facial reconstruction [13].

Complications such as flap failure, infection, wound dehiscence, and hardware exposure are well-documented in the literature and may necessitate secondary revision procedures [14]. Our patient developed hardware exposure nine months postoperatively but had a viable flap and no evidence of malignancy. He underwent successful secondary revision and remains in good health.

The psychosocial impact of orbital exenteration and midface reconstruction should not be understated. Patients may experience diplopia, facial disfigurement, and body image concerns, necessitating psychological support and realistic preoperative counseling.

This case underscores the importance of individualized, multidisciplinary care for advanced facial skin malignancies, combining oncologic resection with innovative, patient-specific reconstructive strategies to restore function and quality of life.

## Conclusions

Recurrent periocular BCC with deep orbital and midfacial invasion poses significant management challenges, often necessitating radical surgical excision when MMS and radiation monotherapy are not available or insufficient. Invasive disease involving bone and critical structures requires a multidisciplinary approach that integrates oncologic resection, advanced imaging, and individualized reconstructive planning. In this case, MMS was not available, and radiation therapy alone could not ensure adequate disease control.

Reconstruction with a split calvarial bone graft and radial forearm free flap provided a durable and anatomically appropriate solution for the complex midfacial and orbital defect. This approach balanced functional preservation with structural integrity and cosmetic considerations. Postoperative complications such as hardware exposure may still arise and necessitate revision surgery, reinforcing the importance of long-term follow-up.
